# A Mobile App With Multimodality Prehabilitation Programs for Patients Awaiting Elective Surgery: Development and Usability Study

**DOI:** 10.2196/32575

**Published:** 2021-12-30

**Authors:** Tianyu Wang, Philip R Stanforth, R Y Declan Fleming, J Stuart Wolf Jr, Dixie Stanforth, Hirofumi Tanaka

**Affiliations:** 1 Department of Kinesiology and Health Education The University of Texas at Austin Austin, TX United States

**Keywords:** mobile app, prehabilitation, perioperative care, rehabilitation, surgery, perioperative, elective surgery, mobile health, health applications, health apps

## Abstract

**Background:**

Complying with a prehabilitation program is difficult for patients who will undergo surgery, owing to transportation challenges and a limited intervention time window. Mobile health (mHealth) using smartphone apps has the potential to remove barriers and improve the effectiveness of prehabilitation.

**Objective:**

This study aimed to develop a mobile app as a tool for facilitating a multidisciplinary prehabilitation protocol involving blood flow restriction training and sport nutrition supplementation.

**Methods:**

The app was developed using “Appy Pie,” a noncoding app development platform. The development process included three stages: (1) determination of principles and requirements of the app through prehabilitation research team meetings; (2) app prototype design using the Appy Pie platform; and (3) app evaluation by clinicians and exercise and fitness specialists, technical professionals from Appy Pie, and non–team-member users.

**Results:**

We developed a prototype of the app with the core focus on a multidisciplinary prehabilitation program with accessory features to improve engagement and adherence to the mHealth intervention as well as research-focused features to evaluate the effects of the program on frailty status, health-related quality of life, and anxiety level among patients awaiting elective surgery. Evaluations by research members and random users (n=8) were consistently positive.

**Conclusions:**

This mobile app has great potential for improving and evaluating the effectiveness of the multidisciplinary prehabilitation intervention in the format of mHealth in future.

## Introduction

Although surgery is often an essential part of the treatment of many diseases, especially solid organ malignancies [1], postoperative recovery remains suboptimal owing to the substantial stress responses induced by surgical trauma [2]. Major surgery is associated with up to a 40% reduction in functional capacity [3], which places patients at an elevated risk for postoperative complications [4]. Globally, approximately 310 million major surgeries are performed every year, with up to 15% of patients experiencing serious postoperative morbidity [5]. Therefore, we deem that improving the physical capacity and resiliency of these patients is critical for improving postoperative recovery. Therefore, this has the capacity to save billions of dollars in health care costs [5].

Traditionally, interventions to improve physical function (ie, rehabilitation) have focused on activities during the postoperative period to improve physical function. However, many patients are unable to perform an effective rehabilitation program owing to underlying frailty and complications from surgery [6]. More recently, there has been a focus on performing interventions prior to surgery—a process referred to as prehabilitation [6]. Prehabilitation aims to enhance physiologic reserve prior to the predictable injurious effects of surgery. It involves medical optimization combined with exercise, nutrition, and psychological programs with the aim of enhancing the overall functional capacity of the patients, so that they can better withstand the physical and mental stressors associated with undergoing a complex surgical procedure [6] and thereby minimize postoperative complications [7]. The typical time frame for a prehabilitation program is only ~4-6 weeks, making intense exercise preferable for achieving effective gains in physical capacity. However, high-intensity exercise may in turn result in a low adherence to the program [8] and is not feasible for many frail individuals [9]. Moreover, prehabilitation exercise programs have most frequently emphasized cardiovascular exercise rather than including resistance training. Even when resistance training exercises are included, there is often low adherence to such a program owing to its challenging nature [8], even though resistance training is crucial for improving muscle strength and will often predict a lower incidence of postoperative complications [10]. Additionally, the largest barrier to participating in prehabilitation reported by patients has been a lack of transportation (ie, arranging transportation and finding or paying for parking) and convenience [8] making any attempt at effective supervision of a multidisciplinary program much more difficult.

We have evaluated a 4-week multidisciplinary prehabilitation program that includes a blood flow restriction (BFR) exercise combined with the daily consumption of a sports nutrition cocktail (including whey protein, creatine monohydrate, and L-citrulline) for patients with abdominal cancer undergoing elective surgery [11]. This program not only elicited a high adherence rate in resistance training (ie, 95%) but also significantly improved functional capacity and lean mass [11]. As the next logical step to extend our research effort, we decided to implement and adapt the multidisciplinary prehabilitation program into a mobile app. Digital health is emerging as a critical assistant in health management and health care because of its cost-effectiveness and high penetration in populations [12-15]. Among various types of digital health interventions, using a mobile health app as a home-based strategy has the potential to positively influence self-efficacy and empowerment of patients [16], and to overcome the biggest barrier—transportation [8]. Although there are more than 50,000 health apps on the market, few prehabilitation apps currently exist, and these are not suitable for multi-modality prehabilitation interventions. Accordingly, the major aim of this study was to develop a mobile app incorporating our prehabilitation program to reduce barriers to patient participation in a prehabilitation program and to increase outreach to a larger population to further validate current prehabilitation protocol.

## Methods

### Methods Overview

The study was brainstormed by a series of multidisciplinary research team meetings. The 7-person team consisted of an exercise physiologist, 2 fitness specialists, a surgical oncologist, a professor in surgery and perioperative care, a PhD nutritionist, and an exercise physiology graduate student. In this study, the team meetings involved two stages: project planning and mobile app evaluation and modifications. In the first stage, the team met 3 times within 2 months to determine the principles, timelines, budget, and website for developing the app. After we obtained the app prototype, the team met 3 times within a month and a half to evaluate, modify, and reevaluate the app. Each meeting lasted 1 hour. Because the schedule of the surgical oncologist fluctuated on the basis of patient appointments, there was no set time for the meeting.

Along with the team meeting, the development process involved three stages: (1) determination of principles and requirements for the app; (2) prototype design; and (3) evaluation of the developed product. This prehabilitation app was developed through the no-code App Builder Appy Pie.

### Prehabilitation Protocol Implemented in the App

The core of this app is the 4-week presurgical BFR exercise and sport nutrition supplement intervention based on a previous program developed and trialed by our group [11]. BFR is a cutting-edge training modality that works by restricting blood flow through the veins by using compression devices similar to traditional blood pressure cuffs [17]. Even if exercises are performed at low to moderate intensity, increases in muscle mass and muscle strength are significant [18], similar to those of high-intensity, heavy resistance training [19]. BFR training has been used safely and effectively in older individuals and physically limited populations [20-23], including presurgical patients with cancer [11]. The participants (mean age 64.9 years, SD 9.8 years) in our previous study reported little difficulty (mean score 1.6, SD 0.6) and high enjoyment (mean score 5.2, SD 0.5) on a standardized 7-point scale when performing the BFR exercise. No adverse events were reported to be associated with the BFR training [11].

### Determination of Principles and Requirements

By having the meetings at the first stage, we agreed upon the following principles for the development of this app:

1. Target population: patients undergoing elective surgery, especially those who are frail and have a short presurgical period during which they might be able to undergo training (eg, patients with cancer who are awaiting elective surgery).
2. Ease of use: since many patients undergoing surgery are older patients who often have concerns about their lack of technological skills [15], we decided to follow a simple user interface design with detailed plain-language instructions.
3. Application of behavior change techniques: implementing strategies such as self-monitoring, social support, feedback, and motivation aims to maintain or even improve adherence rates in the context of mobile health and thus promotes the effectiveness of the intervention.
4. Establish a communication system between the investigative team and patients: even though the patients rely on the app throughout the 4-week intervention, we still need to maintain a constant connection with patients by means of web-based meeting technology and provide options for patients to reach out to exercise professionals.

**Figure 1:**
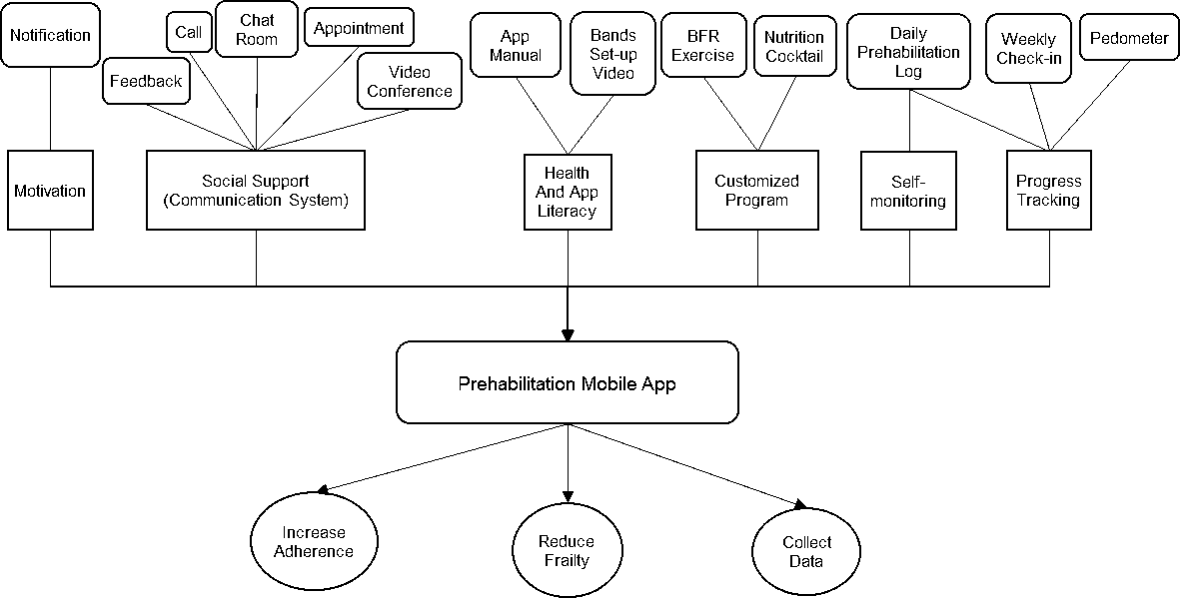
Framework of develoment of the cell phone app for prehabilitation. BFR: blood flow restriction.

Based on these principles, we determined that our requirements (Figure 1) for the app should include the customized BFR exercise and nutrition program, the system of communication with the exercise specialist (instant user feedback, the appointment reservation system, videoconference), support group chat room, pedometer, motivational reminders, and progress-tracking. These principles and feature requirements of the app were confirmed on the basis of the experience from our previous study, literature review, and suggestions from nutritionists, the exercise physiologist, and clinical oncologists in our team.

### Prototype Design

We initiated the design of the app with technical support from Appy Pie, a no-code app builder, after confirming the app requirements. Choices included the following: various interface designs; formats for inserted documents (eg, .docx and .pdf); embedded websites, pictures, and videos in the app; and in-app features (eg, pedometer, notification, login, appointment reservations, and videoconference) to meet our requirements. Taking advantage of the platform and following the framework of the app development (Figure 1), we decided to include the following: BFR exercise instructional videos and nutrition shake instructions to help patients easily follow the prehabilitation program; a daily prehabilitation log and weekly check-in forms to track patient progress; assistance resources including “app manuals,” a “BFR band placement video” to improve patients’ ability to effectively use the BFR bands; and a “user satisfaction survey” to help guide future modifications to the prehabilitation program and the app. In-app functions such as “about us,” “call,” “feedback,” “appointment,” “videoconference,” and “notifications” were also included with the purpose of enhancing engagement and adherence to the e-prehabilitation program. Additionally, surveys and questionnaires were created using Google Forms, which were embedded in the app as an “outer-platform feature.” This was done for data collection as it can link with spreadsheets, allowing responses to be automatically updated once the patients submit responses.

### Evaluation

At the end of the design stage, the app was demonstrated in several team meetings, which led to further modifications. Afterward, we requested a technical evaluation from app development professionals employed by the Appy Pie development team. Finally, we created an app evaluation questionnaire including 6 items on the scale ranging from 1 to 5 (1=strongly disagree, 5=strongly agree) with Google Forms, which allowed the responses to be automatically input in a linked spreadsheet. The questionnaire covered multiple aspects, including engagement, functionality, aesthetics, information, and subjective quality questions extracted from the mHealth App Usability Questionnaire [24]. The questionnaire shown in the table below has been added in the revised manuscript (Table 1). We distributed the app test link along with the survey through email in a convenience sample and the subjects voluntarily filled out the survey within a week. No incentives were offered. Finally, we collected 8 responses in total.

For data analyses, we mainly focused on the descriptive statistics (ie, mean and SD values) of the Likert scale questions rather than the statistical analysis because of the sample size and the lack of control group.

**Table 1:** The app evaluation questionnaire for non–team members (n=8).

Questions	Scale
I am satisfied with this app.	0-5^a^
This app has all the functions and capabilities I expect.	0-5
The information in the app was well organized.	0-5
I like the interface of the app.	0-5
The app was easy to use.	0-5
What’s your favorite feature in this app?	Text

^a^0=strongly disagree; 5=strongly agree. The same principle applies for all grading scales of 0-5 points.

## Results

### Results Overview

**Figure 2:**
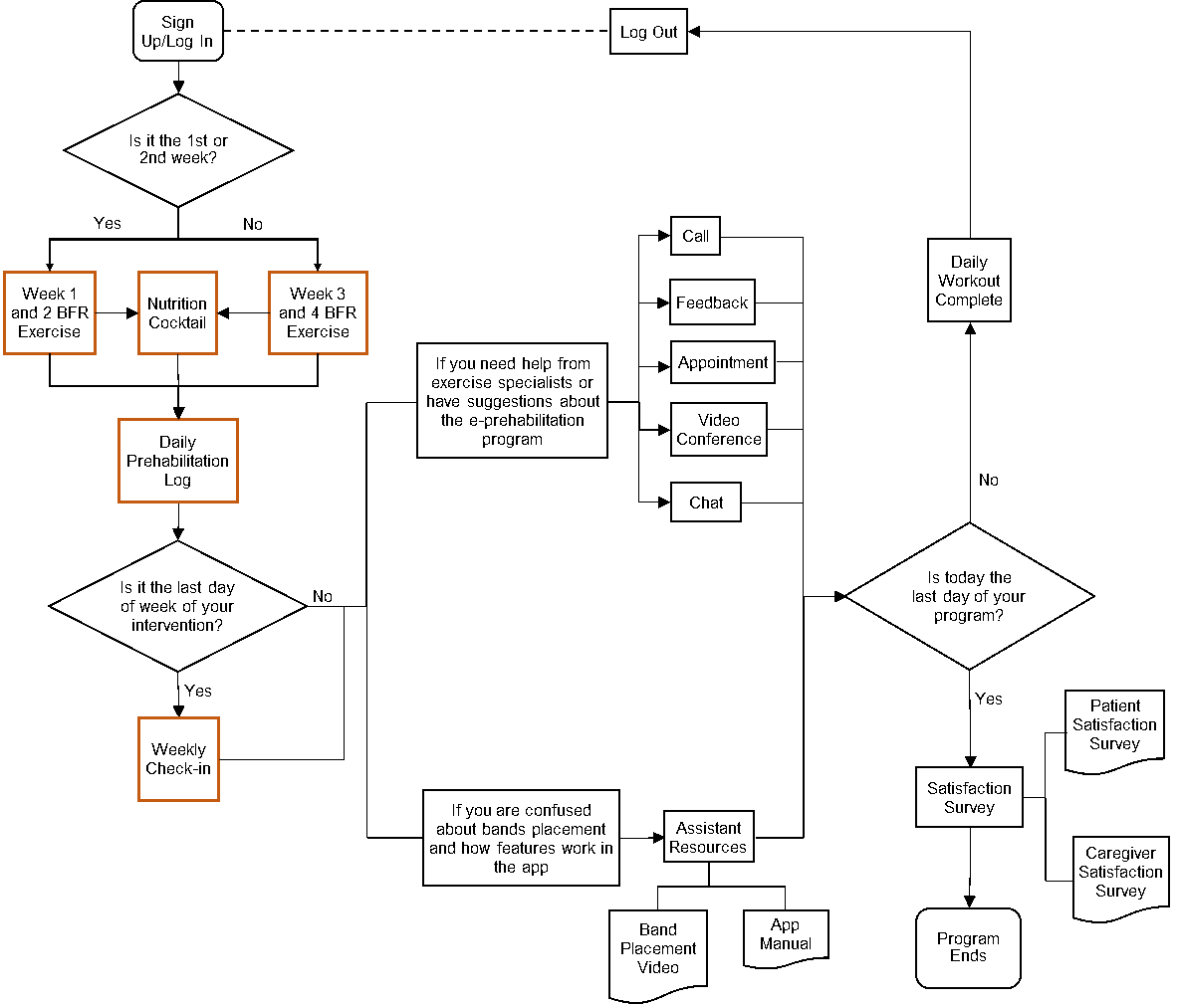
Flowchart of the prehabilitation app. BFR: blood flow restriction.

Following the design principles and requirements, the prototype of the app was developed and named “UT Prehab” [25]. The flow of the app is illustrated in Figure 2. UT Prehab is composed of the core features including BFR exercise, nutrition cocktail instruction, daily log, and weekly check-in, along with accessory features including “about us,” “exercise emergency call,” “feedback,” “appointment,” “videoconference,” “app manual,” “band placement,” “user satisfaction survey,” and “notifications.”

### Display

**Figure 3:**
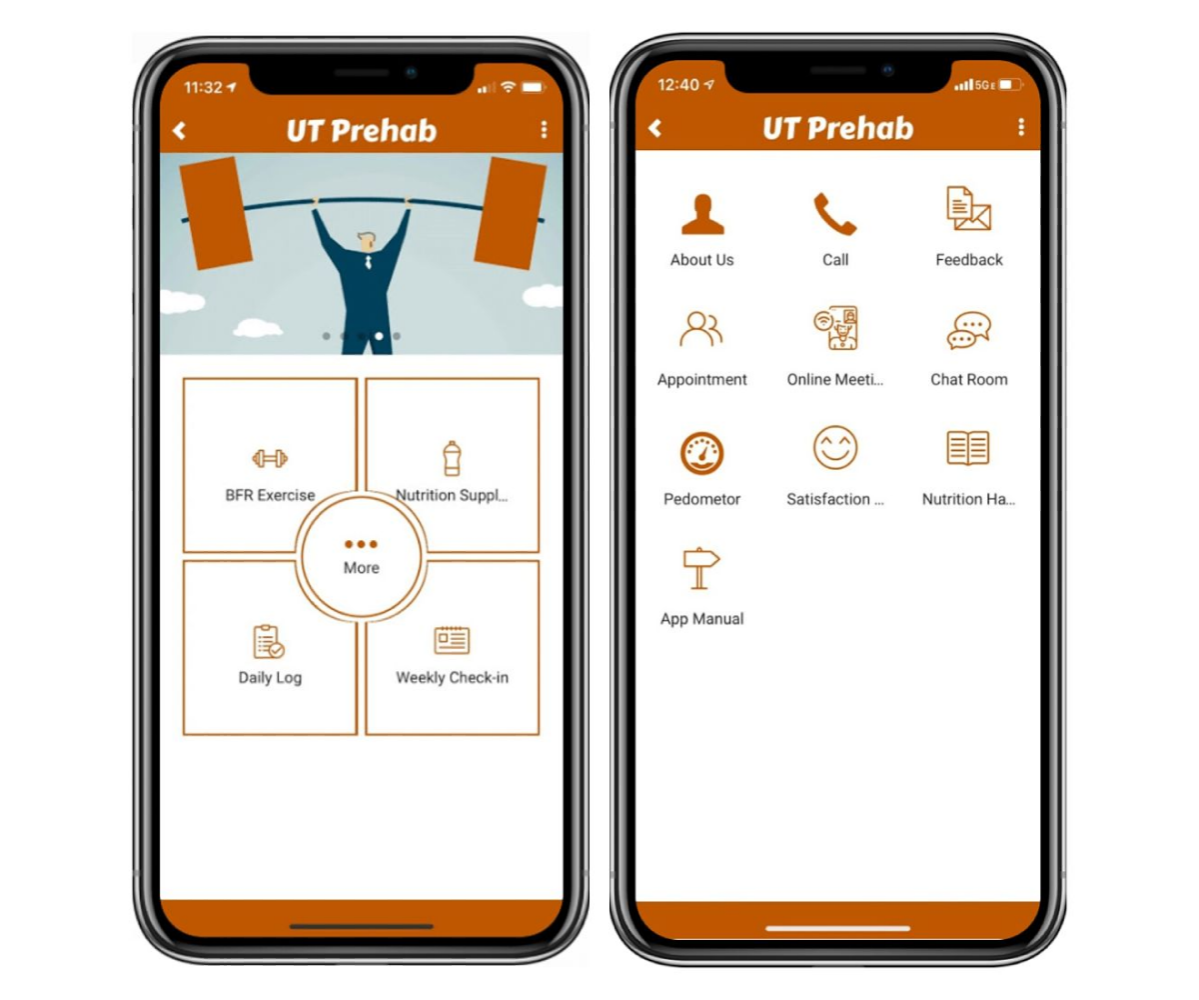
Overview of the home page and accessory features of the prehabilitation app.

The type of display on the home page is “fixed matrix,” which is a simple and key point–stressed design (Figure 3). There are only four main options shown on this page: “BFR exercise,” “Nutrition supplements,” “Daily log,” and “Weekly check-in.” These are core interventions and assessments, as well as the compulsory tasks for the user to complete. The “More” button circled around by the 4 core tasks offers accessory features to assist users to have a better participation experience and to gradually get accustomed to the use of the app.

### Features

#### BFR Exercise and Nutrition Cocktail

**Figure 4:**
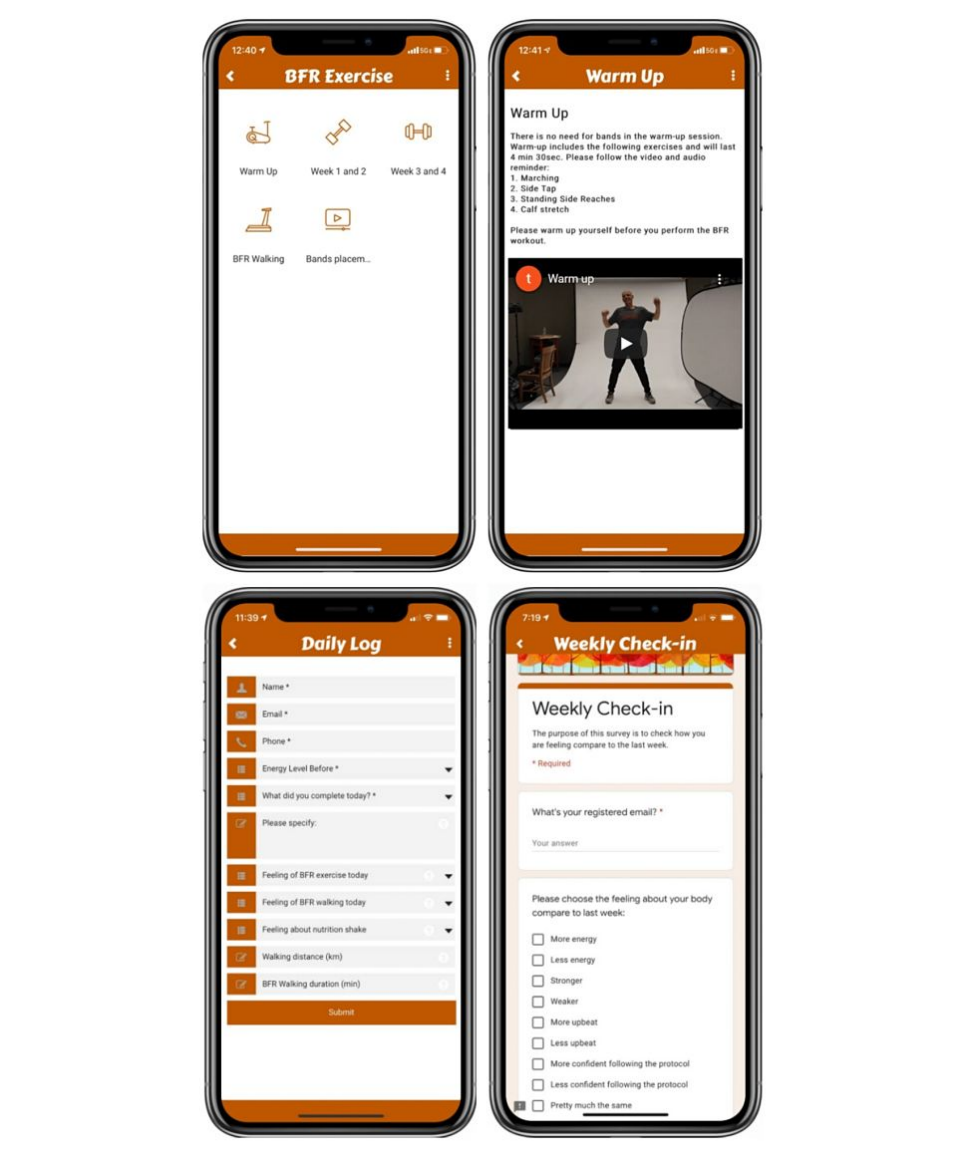
Examples of the blood flow restriction exercise, daily log, and self-report questionnaire features in the UT Prehab app.

“BFR exercise” was composed of five sections: “Warm-up,” “Weeks 1 & 2,” “Weeks 3 & 4,” “BFR walking,” and “Band placement” (Figure 4). Except for “BFR walking,” the other four sections included text instructions and exercise videos with audio instructions and background music. The videos of BFR resistance training were created and published on YouTube [26]. The text instructions were also provided in the BFR walking section. In addition, the “Bands placement” embeds a YouTube video created by B Strong, LLC, which instructs users to set up and inflate the BFR bands on their arms or legs on their own.

The “Nutrition supplements” page includes the directions for formulating and consuming a nutrition shake. The components of the nutrition shake and the corresponding functions are also provided along with the directions for the participants to learn more about the prehabilitation program.

#### Daily Prehabilitation Log

This form includes the exercise duration in minutes, the program they completed, and the participant’s subjective response to the BFR exercise and consuming the nutrition shake (Multimedia Appendix 1). Their response is sent directly to the research team.

#### Weekly Check-in

A short “check-in” survey (Multimedia Appendix 2) created with Google Forms to be filled out on a weekly basis composed of self-reported feelings of energy, strength, upbeat, and confidence levels, compared with the previous week.

#### Chat Room

The “Chat room” is an in-platform feature allowing the patients to chat in a group with other participating patients using the app. They have the option to create their own username or use their real name. Unfriendly messages will be recognized and automatically blocked by the system.

#### Feedback

This form includes personal information of the user as the identifier (ie, email ID and phone number), the preferred way to receive the response (ie, email ID, phone call, or text message), and the detailed information of their feedback request. Their feedback submission will be sent directly to the research team.

#### Appointment

The “Appointment” feature offers an opportunity for the patients to schedule an appointment with exercise specialists. They can check the available time slots and choose dates and times in accordance with their convenience and preference. The options for meeting format include in-person and web-based meetings. If they prefer an in-person meeting, an email with detailed appointment information (eg, location, parking, time, and contact number) will be sent to the patient with the appointment confirmation. The “Videoconference” feature in the app will satisfy their need for a virtual meeting.

#### Videoconference

The “Videoconference” feature embedded in the app provides a convenient platform for both patients and the research team to have web-based meetings as it avoids the need of downloading other virtual meeting apps. This feature allows the participants to have virtual appointments with exercise specialists on the basis of their schedule and other preferences. For example, exercise specialists are able to schedule the meeting in the app and invite the participants through a weblink, message, or email. Patients can join the meeting in the “Videoconference” section or on the login page at the scheduled time by inputting the meeting ID.

#### App Manual

The “App manual” document was implemented within the app. This document not only provides step-by-step directions for each module along with pictures of corresponding user interfaces but also includes the description of the prehabilitation program and the potential benefits of the program observed from our previous study. Even though app training will be provided before patients start the program, the dual installation of the app manual and bands placement video mentioned above will provide assistance for potential issues that the patients may encounter during the home-based intervention.

#### Pedometer

“Pedometer” takes advantage of the GPS system in smartphones and provides the possibility of tracking their spontaneous physical activity in step counts, distance, and calorie expenditure. This function allows participants to set goals on steps, distance, or calories with their basic information (ie, gender, height, and body weight) filled out.

#### User Satisfaction Survey

At the end of the intervention, patients and their caregivers will be asked to fill out the user satisfaction survey and the caregiver satisfaction survey, created using Google Forms (Multimedia Appendix 3). The user survey includes the assessment of feelings (ie, enjoyment, difficulty with the prehabilitation program, ease of use of the app, and information load) and comments or suggestions for improving the e-prehabilitation program. The caregiver survey assesses three topics: overall experience, patient outcomes, and suggestions for improvements.

#### Evaluation Results

**Figure 5:**
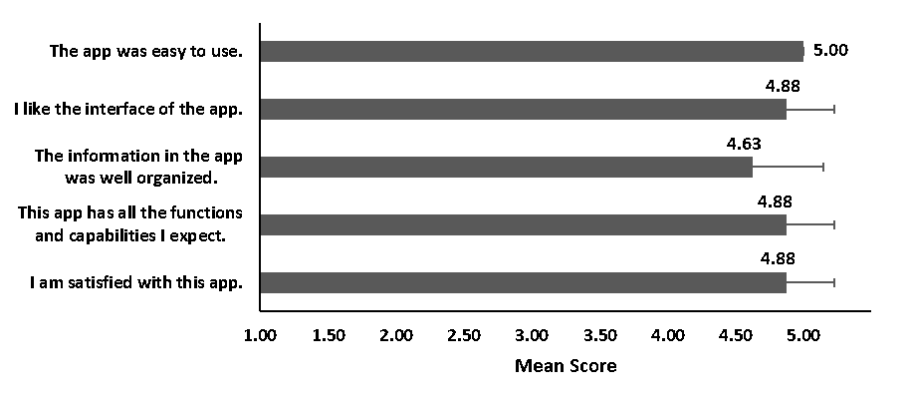
Results of the questionnaire evaluating the usability of the app from non–team members (n=8). Error bars indicate SD values.

Regarding the technical aspect, the Appy Pie professionals assured that the app functioned well at every point. By offering the app test link to the public, the app was tested by 4 female and 4 male participants with ages ranging 21-61 years and a mean age of 37 years (SD 18 years). They graded the app high in simplicity, interface design, organized information, functions, and overall satisfaction (Figure 5) with all average scores higher than 4.5 out of 5.

## Discussion

### Principal Findings

We developed a prototype of a mobile app based on a multidisciplinary sports science–based prehabilitation program that had been previously developed and trialed among patients with abdominal cancer undergoing elective surgery. The prehabilitation app combined the BFR exercise and sport nutrition program with several psychosocial motivating elements and behavior change strategies with the purpose of increasing the effectiveness of the mHealth intervention. The overall satisfaction and usability of the app reported by users are promising.

### Advantages of the Mobile App

The simplicity of the app may reduce the time required for users to participate in the program and improve compliance and adherence [14]. Core task-stressed display and assistant resources embedded in the app may reduce the technology barriers for older or non–tech-savvy patients participating in this eHealth intervention. More specifically, the assistance feature, including a step-by-step manual and BFR band placement tutorial video, will reduce the possibility of causing information or technology overload for the participants and therefore promote their compliance in home-based participation. 

One major problem with currently available mHealth apps is that very few were established with strong research evidence [27,28]. The innovative prehabilitation program implemented in this app was evaluated in our previous study [11], which makes this a more evidence-based program being implemented with this app. Taken together, from the perspectives of the display, embedded program, and assistant feature design, this app provides a simple and evidence-based prehabilitation program that may be applied to a wide range of patients undergoing a variety of treatments.

Consultation with exercise professionals is the most commonly desired feature in exercise interventions [8,29]. A scoping review suggested that the effectiveness of home-based interventions for patients with cancer was largely attributed to the level of attention from qualified exercise professionals [30]. Older patients especially favor being guided during technology-based exercise interventions [31]. Expert consultation was most acceptable or useful to the participants and resulted in higher adherence to the mHealth intervention [32,33]. However, an app that is capable of all these features has, until our program, yet to be developed [33], and home-based exercise is generally unsupervised once the individual leaves the facility in which the exercise was prescribed [30]. Thus, the feedback, appointment, and videoconference features in this app offer the opportunity for patients to receive professional advice and supervision from exercise specialists through their preferred mode of communication, including SMS text messages, telephone calls, email, and in-person or video-based face-to-face meeting. These features allow patients to access the exercise professionals at any point in the program and to maintain communication with exercise specialists even while performing home-based interventions. In this sense, our app has the potential to facilitate the exercise professional–patient relationship and motivate patients to engage in and adhere to the home-based e-prehabilitation program.

Despite the high adherence rate that we observed in our previous study [11], additional barriers may affect interventions in mHealth format. Behavior change techniques used to promote physical activity are one of the main factors affecting engagement with physical activity apps [34]. The behavior change techniques used in this app include self-monitoring, customized intervention, social support, and motivations. The daily prehab log serves as a self-monitoring tool for patients to keep track of their compliance with the program. The pressures inflated in BFR bands are customized by healthy level, extremities’ circumferences, and exercise intensity the participants choose (ie, low, moderate, or high).

Social support (eg, support from friends, family members, and neighbors) is another key construct for enhancing adherence to exercise interventions among patients with cancer [7,35,36] as well as a critical element in home-based interventions [36] and self-management mHealth app–based interventions [13]. A qualitative interview study demonstrated that social support positively influences engagement in physical activity among older adults with abdominal cancer [7]. This app establishes a full social support system by means of “video conference,” “call,” “feedback,” “appointment,” and “chat room” features. Additionally, the “chat room” feature provides the possibility for the patients to form a web-based peer network in which patients are able to encourage each other and share their feelings and stories as a community, which may positively influence their psychological status, engagement, and adherence to the program. Taken together, the interventional features of this app highlight the potential of meeting individualized patient needs, providing motivation, enhancing patient engagement and adherence, and facilitating human interaction. These themes are considered the determinants for the effectiveness of a mobile intervention to support surgical patients [37].

### Limitations

The main limitation of this study is the lack of involvement of patients with abdominal cancer. There are a number of reasons that we could not involve the target patient population. First, this study suffers from limited budget and time. Second, patients with abdominal cancer have a limited time frame before surgery to test the app and provide detailed feedback. Instead, the two experienced clinical oncologists in our team provided a fair number of suggestions to accommodate this app to meet the needs of patients with cancer. For example, the patients might want to call the team member immediately, and we provided the one tap phone call function. They will need professional advice on exercise, and we provided the web-based meeting, feedback, and appointment features. Therefore, we would expect our app to be ready for clinical use to a certain degree. Another limitation is that the small screen on the smartphone makes it difficult for older people to perform the exercise by watching the video. However, the audio instruction and count-down timer were implemented in the video. Once participants get familiar with the exercise regimes, performing the exercise only with audio instruction is possible. If conditions allow, the exercise video can be projected to a larger screen for convenience. In the future, usability and acceptability evaluations should be conducted among the different populations that will be involved in the prehabilitation process (eg, target patients, caregivers, and health care providers). Final modifications will then be made by gathering multi-party opinions before the app is publicly available. The end-product is expected to meet multi-party needs and fit well in the clinical workflow with the support of exercise professionals. We plan to initiate a multicenter research trial by using this app with the purpose of expanding the study population and validating this prehabilitation program.

### Comparison With Prior Work

To the best of our knowledge, UT Prehab is the first prehabilitation app combining evidence-based BFR exercise and sport nutrition programs. There are currently only 4 apps specifically focusing on prehabilitation in the Apple App Store and Google Play. “Craetus Prehab (Craetus)” was designed in an attempt to help prepare patients for cancer treatment by tracking aerobic exercise, mindfulness, and nutrition. However, the programs in “Craetus” are not specific and are only available in the United Kingdom. “Prehab (Eurecat)” is used at Hospital Clinic de Barcelona. It was designed to prepare the patient for abdominal surgery with general instructions about nutrition, mindfulness, and exercise. Access to the app is limited, in that it is granted only by collaborating institutions. Further, the effectiveness of the program employed by the app has not yet been validated, thus limiting the evidence of its potential effectiveness. The “PeerWell” and “Exphy Surgery” apps focus on pain control, but they are limited to musculoskeletal surgery and are prescription-only. None of these apps provide patients undergoing surgery with a customized exercise and specific nutrition program with the support of exercise specialists. UT Prehab is unique, in that it utilizes innovative multidisciplinary prehabilitation interventions and provides a highly interactive environment.

### Conclusions

In this study, we developed a prototype of a mobile app, with the aim to implement a multidisciplinary prehabilitation program for patients who will undergo elective surgery. With the characteristics of simplicity, a validated and scientifically sound prehabilitation program, assistance of recognized behavior change techniques, and communication with the professional prehabilitation team, this app has the potential to positively affect the effectiveness of future e-prehabilitation interventions. Usability and acceptability evaluation by the targeted population and other relevant individuals (ie, caregivers, exercise specialists, and health care providers) will be the next step prior to its use in multicenter studies.

## Appendix

Multimedia Appendix 1 Daily Exercise and Nutrition Log.

Multimedia Appendix 2 Weekly check-in.

Multimedia Appendix 3 Satisfaction survey.

## Abbreviations

BFR: blood flow restriction
mHealth: mobile health
