# Supplementary material for: A Mobile App With Multimodality Prehabilitation Programs for Patients Awaiting Elective Surgery: Development and Usability Study
Source: JMIR Perioper Med. 2021 Dec 30;4(2):e32575. doi: 10.2196/32575 (PMC8759016; doi:10.2196/32575)
Supplement: Multimedia Appendix 1 [file periop_v4i2e32575_app1.docx]

## Multimedia Appendix 1. Daily Exercise and Nutrition Log

Date: *MM/DD/YEAR*

1. Exercise duration (min): _____________________________
2. What did you complete today?

50 min BFR Exercise

15 min BFR walking

400 ml nutrition shake

Part of them

None of them

If you chose “Part of them” in the last question, please specify the duration of the exercise (min) and/or volume of nutrition shake you consumed (e.g., 10 min upper body exercise, 100 ml nutrition shake, etc.):

1. How intense do you feel for overall BFR exercise you performed today (Please answer this question only if you performed BFR resistance training today)?

| 0 – at rest | 6 |
| --- | --- |
| 1 – very easy | 7 – very hard |
| 2 – somewhat easy | 8 |
| 3 – moderate | 9 |
| 4 – somewhat hard | 10 – very, very hard |
| 5 - hard |  |

1. How intense do you feel for overall BFR walking you performed today (Please answer this question only if you performed BFR walking today)?

| 0 – at rest | 6 |
| --- | --- |
| 1 – very easy | 7 – very hard |
| 2 – somewhat easy | 8 |
| 3 – moderate | 9 |
| 4 – somewhat hard | 10 – very, very hard |
| 5 - hard |  |

1. How do you like drinking the nutrition shake (Please answer this question only if you take nutrition shake today):

I like it and finish all of it.

I finished but reluctantly.

I could not finish it.

1. Walking distance in pedometer (please answer this question only if you performed BFR walknig today):__________ km
