# Supplementary material for: A Mobile App With Multimodality Prehabilitation Programs for Patients Awaiting Elective Surgery: Development and Usability Study
Source: JMIR Perioper Med. 2021 Dec 30;4(2):e32575. doi: 10.2196/32575 (PMC8759016; doi:10.2196/32575)
Supplement: Multimedia Appendix 2 [file periop_v4i2e32575_app2.docx]

## Multimedia Appendix 2. Weekly check-in

How are you feeling compared with last week? (Please check all options that apply to you.)

More energetic

Less energetic

Stronger

Weaker

More upbeat

Less upbeat

More confident following the protocol

Less confident following the protocol

Pretty much the same
