# Supplementary material for: A Mobile App With Multimodality Prehabilitation Programs for Patients Awaiting Elective Surgery: Development and Usability Study
Source: JMIR Perioper Med. 2021 Dec 30;4(2):e32575. doi: 10.2196/32575 (PMC8759016; doi:10.2196/32575)
Supplement: Multimedia Appendix 3 [file periop_v4i2e32575_app3.docx]

## Multimedia Appendix 3. Satisfaction survey

***User satisfaction survey***

1. How difficult was it to perform exercise and walking with the blood flow restriction bands?

1 (Not difficult at all)

2

3

4

5

6

7 (Very Difficult)

1. How long did it take to get comfortable with the blood flow restriction bands / exercise?

1 (Immediately)

2

3

4

5

6

7 (I never got comfortable)

1. How much did you enjoy exercise with the blood flow restriction bands?

1 (Not at all enjoyable)

2

3

4

5

6

7 (Enjoyed a lot)

1. How easy for you to understand the information displayed in this app?

1 (Very easy)

2

3

4

5

6

7 (Very hard)

1. How much load did you feel while using the app to participate in prehab program?

1 (No load at all)

2

3

4

5

6

7 (Very hard)

1. Which section(s) in the app do you like best? Why?
2. Which section(s) in the app do you dislike most? Why?
3. Do you have any other comments or suggestions for this program?

***Caregiver Satisfaction Survey***

Part A: Overall Experience

1. Rate your overall level of satisfaction with the BFR Training Protocol

Very satisfied

Satisfied

Neutral

Dissatisfied

Very dissatisfied

1. Overall, how did this form of training compare to your family member/partner/ spouses’ previous exercise regimen?

Better

Just as good

Not as good

1. Did your family member/partner/ spouse skip any days of training?

Yes

No

I don’t know.

If yes, please describe why below and how many days:

Part B: Outcomes

1. Please indicate your level of agreement with the following statements regarding your family member/partner/spouse’ training efforts

|  | Never | Rarely | Neutral | Sometimes | Always | Not Sure |
| --- | --- | --- | --- | --- | --- | --- |
| They reached fatigue during every exercise session. |  |  |  |  |  |  |
| The training protocol challenged them. |  |  |  |  |  |  |
| They gave 100% effort to complete this program. |  |  |  |  |  |  |
| They forgot to take the nutrition shake after exercise. |  |  |  |  |  |  |
| They skipped days of training. |  |  |  |  |  |  |
| The exercises kept them engaged. |  |  |  |  |  |  |

Part C: Suggestions for Improvement

5. What did you like most about this training?

6. What aspects of the training could be improved? Please share your ideas about items that were missing, additional items that you would like to see, and how the sessions could have been improved.

7. What types of support would help you improve your adherence to this program? Consider training (information and skills-based) and resources needed.

8. Please share any additional comments**.**
